# Effects of rumen-protective γ-aminobutyric acid additive on lactation performance and serum biochemistry in heat-stressed cows

**DOI:** 10.3389/fvets.2023.1228155

**Published:** 2023-09-22

**Authors:** Yanjing Su, Zhiqiang Cheng, Wengboyang Liu, Tianyou Wu, Wendan Wang, Miao Lin

**Affiliations:** ^1^Bright Farming Co. LTD, Shanghai, China; ^2^College of Animal Science and Technology, Yangzhou University, Yangzhou, China

**Keywords:** rumen-protective γ-aminobutyric acid, heat stress, lactating cows, serum biochemistry, lactation performance

## Abstract

In the context of global warming, heat stress has become one of the major stress factors limiting dairy cattle production. Although many methods have been explored to help cows mitigate the negative effects of heat stress during the hot summer months, maintaining the performance of high-yielding cows under heat stress is still a great challenge. The aim of this trial was to investigate the effect of RP-GABA in the diet on milk yield, milk composition and serum biochemical parameters in heat-stressed cows. Twenty Chinese Holstein cows in early lactation (51.00 ± 4.92 kg milk/d, 71 ± 10.94 d in milk and 2.68 ± 0.73 parities) were included in this experiment and randomly divided into four groups (n = 5/group). The four experimental groups consisted of one control group (0 g RP-GABA/d) and three treatment groups, given 5, 7.5 and 10 g RP-GABA/d of dry matter (DM) per cow, respectively. The results showed that supplementing high-yielding cows with 10 g/d of RP-GABA improved milk protein production but had no effect on the improvement of other production performance, the alleviation of heat stress in cows, or the improvement of immune function and antioxidant capacity. Ultimately, we conclude that the supplementation of 10 g/d RP-GABA to heat-stressed, high-yielding dairy cows can provide a degree of performance enhancement. Furthermore, our study provides some reference for nutritional improvement measures for summer heat stress in dairy cows, especially high-yielding cows.

## 1. Introduction

With ambient temperatures rising by 1.0°C since the 19th century and a further 1.5°C expected between 2030 and 2052, heat stress is a major stressor affecting dairy production in the context of global warming (1). Heat stress is defined as the sum of the internal and external forces acting on the animal, resulting in an increase in body temperature and causing a physiological response (2). When heat stress occurs in cows, they are unable to regulate their body temperature effectively and have difficulty producing normally, resulting in increased body temperature and respiratory rate, reduced rumination time and feed intake, ultimately leading to reduced milk production and reproductive performance (2–6). Although some of the negative effects associated with heat stress can be helped by the provision of feeding facilities such as shades, fans and misters, heat stress remains a serious problem in the dairy industry (7–9). Hence, there is a need to explore new nutritional strategies based on physiological and metabolic adaptations, which can help to help cows resist heat stress (10).

The γ-aminobutyric acid (GABA) is an important functional non-protein amino acid and is the predominant inhibitory neurotransmitter in the central nervous tissue of animals (11, 12). GABA not only has various physiological activities such as sedation, anti-inflammation, appetite regulation and hormone secretion, but also has certain physiological functions such as regulation of body temperature and feed intake (12–15). Under hot environment conditions, a single oral dose of GABA can reduce the body’s core temperature by reducing total heat production (16). GABA can improve animal performance and organism health by increasing feed intake, apparent nutrient utilization and immunity (12, 13, 17, 18). In the hot summer months, feeding GABA to cows can reduce rectal temperature, increase DMI and milk yield, and improve milk protein and lactose concentrations, thereby alleviating heat stress without affecting nutrient digestibility (13). Cheng et al. showed that supplementing heat-stressed cows with rumen-protected GABA improved their immune function and antioxidant activity (17). In addition to this, dietary supplementation with GABA improved nutrient digestibility, growth performance and antioxidant status in heat-stressed beef cows (18). Chen et al. study showed that GABA in synergy with Chinese herbs could effectively modulate the heat stress response in beef cows by improving antioxidant capacity, serum parameters and heat shock protein (HSP) expression (19). Previous studies have shown GABA to be beneficial to heat-stressed cows in terms of feed intake, lactation performance and animal health. However, most of the cows selected for these studies were in mid-lactation rather than at the peak of lactation. The nutritional requirements of high-yielding cows under heat stress conditions are more stringent, but the role of RP-GABA in this is poorly understood.

Hence, we hypothesized that supplementing heat-stressed cows in early lactation with rumen-protected GAGB (RP-GABA) during the summer months will improve lactation performance and body health. Therefore, the aim of this trial was to investigate the effect of supplementing the diet with rumen-protective GABA on lactation performance, serum immunity and antioxidant parameters in heat-stressed cows during lactation.

## 2. Materials and methods

### 2.1. Experimental design and animals

This experiment was conducted in July 2022 at the Jinshan Star II dairy farm in Shanghai, China. All cows used in this study were kept under the permission of the standards of Yangzhou University, the Institutional Animal Care and Use Committee (SYXK (Su) IACUC 2016–0019). Twenty lactating Holstein bovines (51.00 ± 4.92 kg milk/d, 71 ± 10.94 d in milk and 2.68 ± 0.73 parities at the start of the experiment) were included in this experiment and randomly divided into four groups (n = 5/group). The experiment lasted for 48 days as phase 1 (1–12 days), phase 2 (13–24 days), phase 3 (25–36 days) and phase 4 (27–48 days). The four experimental groups consisted of a control group (0 g RP-GABA/d) and three treatment groups, given 5, 7.5 and 10 g RP-GABA/d DM per cow, respectively. The RP-GABA (75% GABA coated by 25% hydrogenated palm oil, 12 h Rumen protection >85%, Jinan Xuze Biotechnology Co., Ltd.) was supplemented to cows using the same method as cheng et al. (20). Specifically, a portion of the TMR was mixed with RP-GABA and fed to the cows first, and then the remaining TMR was supplemented after the cows had finished eating. The TMR was formulated according to the NRC and its composition and nutritional levels are shown in Table 1. All test cows were assigned to the same barn (different segregated areas of the same barn) for management, with a consistent cooling system, free access to fresh water and a 10% refusal diet. Cows were milked 3 times a day at 8-h intervals (6,00, 14:00 and 22:00). The ambient temperature (T) and relative humidity (RT) of the barn were recorded daily (07,00, 14:00 and 21:00) using the thermometer and hygrometer on the barn temperature control and ventilation system. THI was calculated using the following formula: THI = (1.8 × T + 32) - (0.55–0.0055 × RH) × (1.8 × T - 26) (21).

**Table 1:** Composition and nutrient levels of the experiment diet [dry matter (DM) basis %].

Ingredients	Content
Whole corn silage	23.26
Alfalfa (RFV150)	13.92
Corn	22.39
Soybean meal	6.57
Oat	4.49
DDGS	1.75
Corn germ meal	3.50
Flaked corn	7.12
Double low rapeseed meal	2.18
Wheat bran	4.82
Mountain flour	0.89
Beet pulp	3.73
Cotton seed meal	3.73
Premix^a^	0.43
Calcium hydrogen phosphate	0.43
Sodium chloride	0.43
Sodium bicarbonate	0.36
Total	100
Nutrient levels	
CP	17.4
Starch	27.2
NDF	27.8
Calcium	0.91
Phosphorus	0.41
NE_L_ (Mcal/kg)^b^	1.73

DDGS, distillers dried grains with soluble; CP, crude protein; NDF, neutral detergent fiber.

a Per kilogram of premix provide 4,680 mg of Mn as MnSO_4_; 15,800 mg of Zn as ZnSO_4_; 3,040 mg of Fe as Fe_2_ (SO_4_)_3_; 3,605 mg of Cu as CuSO_2_; 150 mg of I as KI; 30 mg of Co as CoCl2; 1,000,000 IU of Vitamin A; 302,000 IU of Vitamin D; 4,650 mg of Vitamin E; 2,000 mg of niacin.

b Predicted values from NASEM, (2021) model.

### 2.2. Sampling and analyses

In the last two days of each experimental phase the feed provided and the remaining feed was weighed to determine DMI. All feed collected was mixed in proportion and subsequently dried and stored in an oven at 65°C for 48 h until its chemical composition was analyzed according to the previous method (20).

Milk production was measured on the last day of each experimental phase and milk samples were collected from the morning, mid and evening milkings using a meter (DeLaval, Sweden) and milk samples were synthesized according to 4:3:3. This was then submitted to Dairy One Cooperative Inc. (Shanghai, China) for milk composition analysis within 12 h.

On the same experimental day as the milk yield calculation, a blood sample (15 mL) was collected from the tail vein of the cow prior to morning feeding using a syringe. It was immediately placed in a 15 mL tube, allowed to clot and then centrifuged at 3000 g for 15 min at 4°C to collect the serum. The serum samples were stored in liquid nitrogen for rapid cooling and then stored at - 80°C for later biochemical analysis. The superoxide dismutase (SOD), glutathione peroxidase (GSH-PX), total antioxidant capacity (T-AOC), and malondialdehyde (MDA) concentrations were determined using a colourimetric kit (Nanjing Jiancheng Bioengineering Institute). Alanine aminotransferase (ALT), aspartate aminotransferase (AST), alkaline phosphatase (ALP), total protein (TP), triglyceride (TG) and nonesterified fatty acid (NEFA) were detected using a biochemical analyzer (Mindray; BS-420) and the kits (BioSino Bio-Technology & Science, Bejing, China) mentioned. IgG, tumor necrosis factor alpha (TNF-α), interleukin 6 (IL-6), heat shock protein 70 (HSP-70), neuropeptide Y (NPY), corticotrophin releasing hormone (CRH), adrenocorticotropic hormone (ACTH), thyroid stimulating hormone (TSH) and cortisol (COR) were detected using an Elisa kit (Beijing Sino-Uk Institute of Biological Technology, Bejing, China; intra-assay CV ≤ 5%; interassay CV ≤ 10%).

### 2.3. Statistical analysis

Data obtained from the trials were subjected to a two-way ANOVA using the General Linear Model (GLM) procedure of SAS 9.4 (SAS Institute Inc., Cary, NC, United States). The model included the amount of supplemented RP-GABA, the experimental phase and the main effects of its interaction. Each cow was used as the experimental unit. Differences between means were tested using the least significant difference (LSD) method and significance was declared at *p* < 0.05.

## 3. Results

### 3.1. THI and determination of heat stress

**Figure 1:**
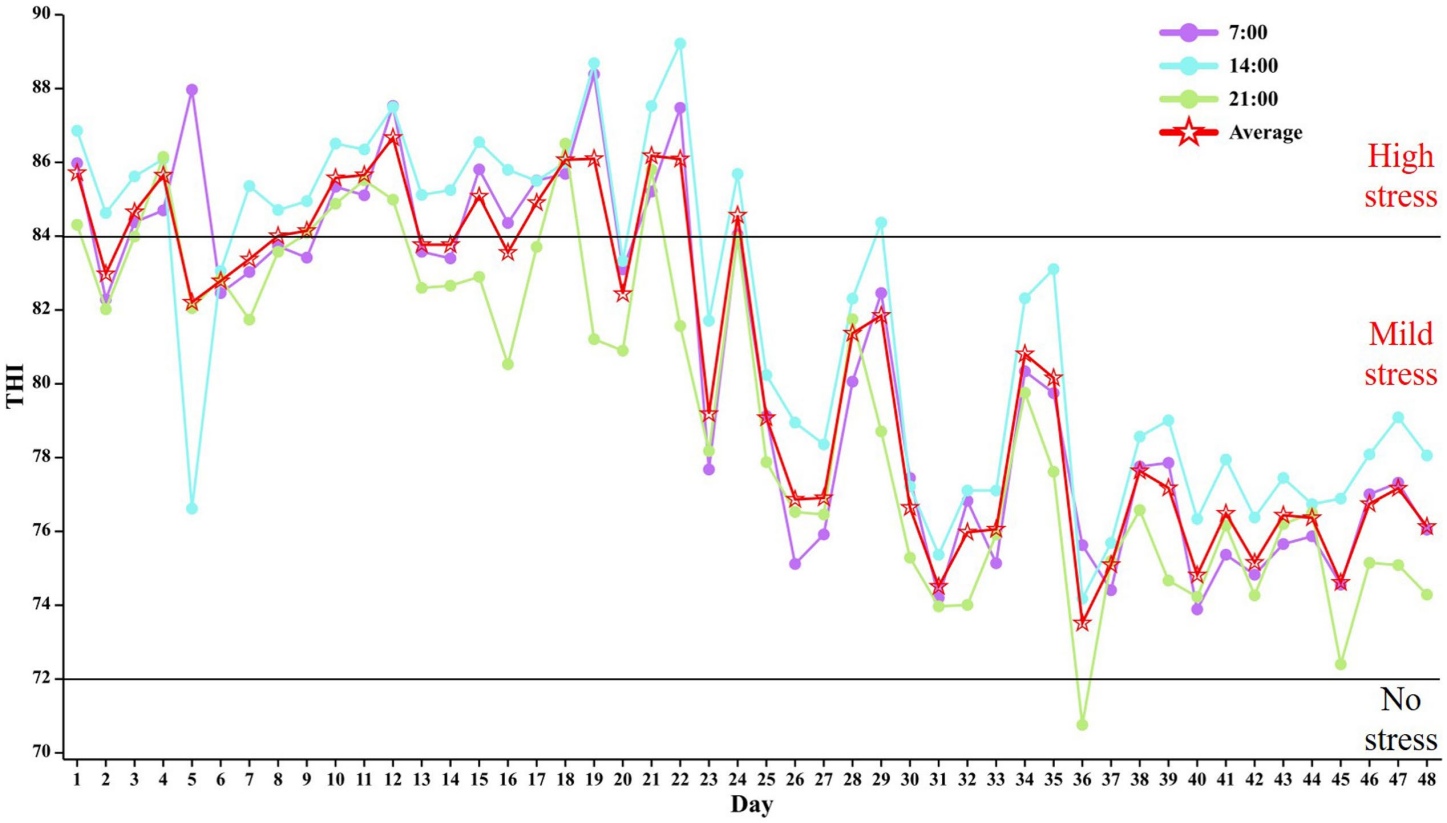
Temperature and humidity index (THI) in the barn during the experiment.

The mean THI was 80.66 (73.87 to 80.6) at 07:00 h, 81.84 (75.6 to 83.3) at 14:00 h and 79.48 (74.8 to 80.8) at 21:00 h (Figure 1). As the trial progressed, there was a decreasing trend in the average temperature of THI in the barn at each phase (phase 1: 84.43; phase 2: 84.29; phase 3: 77.79; phase 4: 76.14). The rectal temperature in heat-stressed cows decreased with the test phase (*p* < 0.001) but was not affected by RP-GAGB supplementation (*p* > 0.05).

### 3.2. Milk yield and Milk compositions

As shown in Table 2, The interaction between the addition of RP-GABA and the phase of trial did not affect the productive performance of the cows (*p* > 0.05). Supplementation with RP-GABA did not affect (*p* > 0.05) cow DMI and cows in the phase 1 of trial had the lowest (*p* < 0.001) DMI. Under RP-GABA treatment conditions, milk protein production was higher in cows supplemented with 10 g/d RP-GABA than in the control and 5 g/d RP-GABA treatment groups (*p* < 0.05). However, the addition of a low dose (5 g/d) of RP-GABA to the ration reduced milk fat production compared to the control (*p* < 0.05).

**Table 2:** Effect of RP-GABA on the performance of heat-stressed cows.

Item	Treatment				Time				SEM	*p*-value		
	CON	5 g/d	7.5 g/d	10 g/d	1	2	3	4		Treatment	Time	Treatment*Time
DMI	26.48	27.45	26.8	28.12	25.08 ^b^	27.14 ^a^	27.86 ^a^	28.77 ^a^	0.28	0.189	<0.001	0.052
Production (Kg/d)												
Milk	49.83	51.93	51.00	52.94	55.51^a^	51.15^b^	51.11^b^	47.62^b^	0.60	0.328	<0.001	0.973
4% FCM^1^	49.58^xy^	48.07^y^	48.70^xy^	51.18^x^	52.42^a^	48.00^b^	48.19^b^	48.93^b^	0.46	0.119	0.003	0.788
ECM^2^	46.17	44.61	44.72	47.20	48.50^a^	44.24^b^	44.77^b^	45.20^b^	0.45	0.150	0.005	0.800
Fat	1.75^x^	1.59^z^	1.62^yz^	1.74^xy^	1.75^a^	1.58^c^	1.62^bc^	1.73^ab^	0.02	0.016	0.010	0.817
Protein	1.48^y^	1.46^y^	1.53^xy^	1.58^x^	1.60^a^	1.49^b^	1.45^b^	1.49^b^	0.01	0.014	0.002	0.835
Lactose	2.55	2.58	2.52	2.67	2.74^a^	2.52^b^	2.51^b^	2.53^b^	0.03	0.417	0.040	0.942
Total solids	6.19	6.09	6.10	6.37	6.56^a^	6.01^b^	5.97^b^	6.21^ab^	0.06	0.399	0.006	0.676
Milk component (%)												
Fat	3.56^x^	3.08^y^	3.19^y^	3.32^xy^	3.17^b^	3.10^b^	3.19^b^	3.68^a^	0.05	0.006	<0.001	0.995
Protein	2.99^x^	2.82^y^	3.01^x^	3.01^x^	2.89^b^	2.92^b^	2.85^b^	3.15^a^	0.02	0.001	<0.001	0.999
Lactose	5.14^x^	4.98^y^	4.93^y^	5.05^xy^	4.94^b^	4.92^b^	4.91^b^	5.32^a^	0.02	0.003	<0.001	0.997
Total solids	12.50^x^	11.77^y^	11.98^y^	12.11^xy^	11.82^b^	11.75^b^	11.72^b^	13.06^a^	0.08	0.021	<0.001	0.994
MUN (mg/100 mL)	15.05	14.68	14.86	15.32	16.28^a^	16.04^a^	12.41^b^	15.10^a^	0.22	0.783	<0.001	0.939

Means values within a row with different letters were significantly different (*p* < 0.05). ^xyz^Represent comparisons between RP-GABA treatment groups. ^abc^Represent comparisons between different trial phases.

1 4% FCM = 0.4*Milk (kg/d) + 15*Fat (kg/d).

2 ECM = 0.327*Milk (kg/d) + 12.95*Fat (kg/d) + 7.20*Protein (kg.d). ^*^Represents the interaction of two factors (treatment and time).

**Figure 2:**
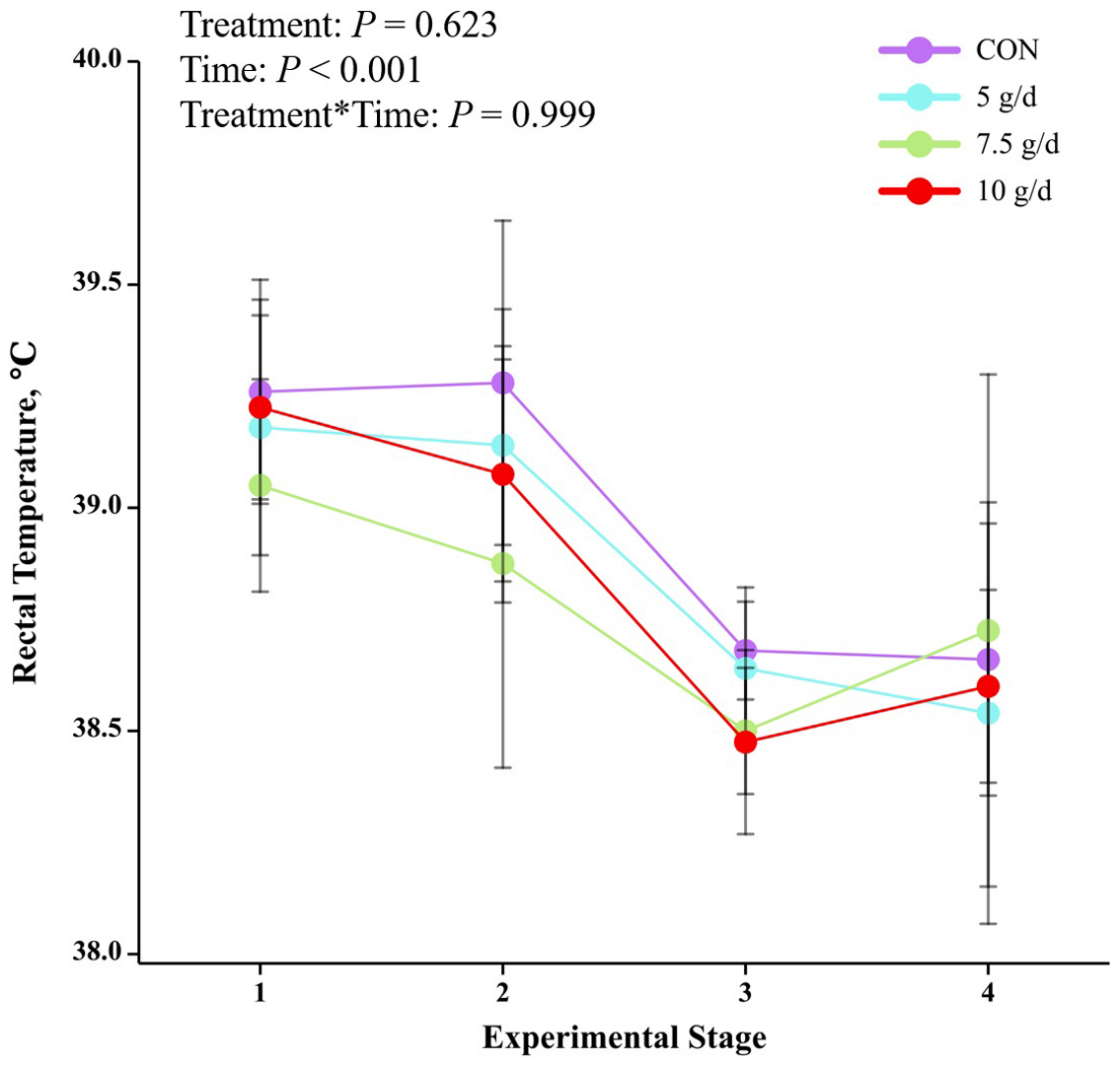
Effect of supplemental RP-GABA on rectal temperature in heat-stressed cows.

Under the time conditions of the trial, cows had the highest yields of lactation, 4% ECM, FCM, milk fat, milk protein, lactose, and total solids in milk in the phase 1 of the trial (*p* < 0.05). However, milk fat, milk protein, lactose and total solids in milk were highest in the phase 4 of the trial (*p* < 0.001). In contrast, MUN levels were lower in the phase 3 of feeding compared to the other phase (*p* < 0.001) (see Figure 2).

### 3.3. Blood indicators

The interaction between the addition of RP-GABA and the duration of the test affected blood MDA (*p* < 0.05) levels (Table 3), but had no effect on blood TP, ALT and NEFA (*p* > 0.05).

**Table 3:** Effect of RP-GABA on blood metabolites in heat-stressed cows.

Item	Treatment				Time				SEM	*p*-value		
	CON	5 g/d	7.5 g/d	10 g/d	1	2	3	4		Treatment	Time	Treatment*time
SOD (U/mL)	46.05	49.05	48.67	47.25	56.64^a^	51.76^ab^	47.05^b^	35.58^c^	1.29	0.824	<0.001	0.101
GSH-PX (U/mL)	448.29	401.97	438.22	439.34	503.98^a^	444.30^b^	414.65^b^	364.88^c^	7.06	0.095	<0.001	0.969
TAOC (U/mL)	10.60	9.16	9.43	9.08	11.53^a^	9.64^b^	8.93^b^	8.17^b^	0.30	0.243	0.001	0.714
MDA (nmol/mL)	4.22^y^	4.15^y^	4.71^x^	4.54^xy^	3.32^d^	4.41^c^	4.76^b^	5.13^a^	0.06	0.004	<0.001	0.021
IgG (g/L)	11.16	11.16	11.04	11.06	11.62^ab^	11.84^a^	10.44^b^	10.52^b^	0.18	0.992	0.012	0.206
TNF-α (pg/mL)	58.28	55.88	59.46	59.02	47.13^d^	55.72^c^	62.25^b^	67.55^a^	0.86	0.442	<0.001	0.904
IL-6 (pg/mL)	125.78	124.22	124.99	125.62	100.18^d^	121.53^c^	134.79^b^	144.11^a^	0.71	0.856	<0.001	0.628
HSP-70 (pg/mL)	228.82	235.80	242.02	227.27	181.58^d^	220.72^c^	258.54^b^	273.06^a^	2.20	0.077	<0.001	0.270
NPY (pg/mL)	219.32	215.47	213.94	216.81	183.88^d^	205.78^c^	225.93^b^	249.94^a^	1.61	0.660	<0.001	0.730
CRH (ng/mL)	5.26	4.68	4.81	4.94	4.06^c^	4.69^b^	4.99^b^	5.97^a^	0.10	0.175	<0.001	0.162
ACTH (pg/mL)	22.89	23.23	23.25	22.28	17.13^d^	22.33^c^	24.98^b^	27.22^a^	0.29	0.641	<0.001	0.008
TSH (μIU/ml)	4.80	4.86	4.79	4.95	5.20^a^	4.97^a^	4.73^ab^	4.50^b^	0.07	0.871	0.005	0.618
COR (ng/mL)	67.84	66.22	67.40	67.12	61.22^c^	65.50^b^	68.11^b^	73.75^a^	0.50	0.678	<0.001	0.840
TP (g/L)	85.17	89.68	90.10	87.74	86.37	86.38	91.05	88.89	1.31	0.511	0.533	0.791
AST (U/L)	76.20^y^	85.35^xy^	85.75^xy^	99.44^x^	76.51^b^	82.01^b^	88.51^ab^	99.70^a^	2.12	0.005	0.002	0.971
ALT (U/L)	22.40	23.05	21.30	22.38	20.18	21.90	23.39	23.66	0.55	0.709	0.106	0.647
ALP (U/L)	44.00^xy^	42.35^xy^	37.95^y^	52.69^x^	42.38	39.13	46.15	49.34	1.53	0.015	0.109	0.995
TG (mmol/L)	0.38^y^	0.37^y^	0.40^x^	0.38^y^	0.36^b^	0.38^ab^	0.39^ab^	0.39^a^	<0.01	0.004	0.026	0.933
NEFA (mmol/L)	0.18	0.17	0.18	0.20	0.19	0.18	0.17	0.18	<0.01	0.276	0.434	0.931

Means values within a row with different letters were significantly different (*p* < 0.05). ^xyz^Represent comparisons between RP-GABA treatment groups. abcRepresent comparisons between different trial phases.

^*^Represents the interaction of two factors (treatment and time).

Under RP-GABA treatment conditions, supplementation with 7.5 g/d RP-GABA up-regulated blood MDA and TG levels (*p* < 0.05). In contrast, the addition of 10 g/d RP-GABA to the diet increased blood AST and ALP concentrations (*p* < 0.05).

Under the time conditions of the trial, SOD, GSH-PX, TAOC and TSH concentrations in the blood of cows were highest in the phase 1 of the trial compared to the other phases (*p* < 0.05). In the phase 2 of the trial, IgG levels in the blood of cows were higher than phase 3 and phase 4 (*p* < 0.05). However, MDA, NPY, CRH, ACTH, TNF-α, IL-6, HSP-70, COR, AST, and TG concentrations were higher in cow blood in the phase 4 (*p* < 0.05).

## 4. Discussion

Chinese Holstein cows are very susceptible to heat stress and have a relatively narrow thermal comfort zone due to their cold and heat-tolerant nature (1, 22). In hot conditions, cows are susceptible to heat stress, which can impair their performance and health (1, 23). The Earth’s ambient temperature has increased by 1.0°C since the 19th century and is expected to increase by a further 1.5°C between 2030 and 2052, making managing heat stress more challenging than ever (1, 8). In a previous study, cheng et al. showed that supplementing mid-lactation cows with RP-GABA could alleviate heat stress by reducing the rectal temperature, increasing DMI and milk production and improving milk composition (13, 17). However, it is unclear whether supplementation with RP-GABA has the ability to alleviate heat stress in early lactation cows. Maintaining lactation and body health in heat-stressed cows in early lactation appears to be more challenging than ever due to the stress of the immediate parturition and transition period and the rapid attainment of peak lactation in response to endocrine hormones (24–26). Our hypothesis is that supplementation of RP-GAGB to heat-stressed cows in early lactation during the summer may improve lactation performance and body health by enhancing antioxidant capacity and attenuating the heat shock response. In this study, RP-GAGB supplementation increased milk protein production in cows, although it did not enhance antioxidant capacity or attenuate the heat shock response. These findings may provide a more effective nutritional strategy for maintaining the performance of heat-stressed cows in early lactation.

In summer, cows begin to experience the negative effects of severe heat stress when rectal temperatures exceed 38.9°C (22, 27). In addition, cows begin to experience heat stress when THI > 72 (27, 28). In our study, the maximum THI averaged 81.84 and the minimum THI averaged 79.48, and the majority of the time cows had rectal temperatures above 38.9°C. In addition to this, it was easy to see from the THI and rectal temperatures that cows suffered more severe heat stress in the first two feeding stages due to higher THI. In conclusion, the cows were subjected to heat stress conditions during the experiment and the THI in the barn gradually decreased as the feeding trial progressed.

In general, heat stress reduces DMI and is a major factor that negatively affects the health and production of cows (25, 29). GABA and its agonists may be able to regulate feed intake in some mammals through effects on the central and peripheral nervous systems (13, 17). Cheng et al. showed that DMI tended to increase linearly with increasing levels of GABA supplementation during the hot summer months, possibly because GABA increased feed intake by reducing heat stress in cows (13). GABA can co-express with NPY in the central nervous system and the increased DMI in GABA-supplemented cows may be associated with increased serum levels of GABA and NPY in these cows (12, 30). Differences in DMI may be due to differences in test animal conditions, GABA supplementation levels, ambient temperature and experimental diet (13). Despite being under summer heat stress, the cows used in this experiment were in peak lactation and their lactation and DMI were high enough that increasing their DMI and milk production again through GABA supplementation would be a huge challenge. In addition, in this experiment, cow DMI and serum NPY concentrations were upregulated with increasing time in the experiment. This may be partly due to the down-regulation of THI in cows to promote feeding, and partly due to cows moving out of the transition period into peak feeding, unfortunately, these related hormonal change indicators were not tested. Although we observed that the interaction between the addition of RP-GABA and the duration of the trial did not affect cow performance, excitingly, we found that 10 g/d of RP-GABA treatment up-regulated milk protein production in cows, suggesting that GABA may have the potential to improve milk protein secretion. However, information on the effect of GABA on milk protein synthesis in dairy cows is scarce and further research is needed.

Heat stress activates the hypothalamic–pituitary–adrenal axis in cows, and this neuroendocrine response aims to reduce the negative effects of heat stress. In fact, during HS, the paraventricular nucleus of the hypothalamus produces corticotropin-releasing hormone (CRH) (31, 32). CRH acts on the adrenocorticotropic hormone secretion of the pituitary gland, ACTH, which regulates heat stress in cows by influencing cortisol (31). In addition to the adrenal glands, the thyroid gland is also highly sensitive to temperature and is affected by heat stress imposed by high ambient temperatures (31–33). However, in the present study, GABA did not alter the relevant neuroendocrine hormones (e.g., CRH, ACTH, TSH and COR) in the blood of heat-stressed cows. Surprisingly, the aforementioned endocrine hormone concentrations were affected by the duration of the experiment, which may be due to the complex endocrine changes in cows during early lactation.

Higher DMI and stable lactation in dairy cows require a better antioxidant status, which is reflected by changes in serum metabolites (12, 34). SOD plays an important role in the animal’s antioxidant defense mechanism, while GSH-PX scavenges peroxides and hydroxyl radicals produced during cellular respiratory metabolism (19, 35). T-AOC is the total antioxidant level of various antioxidants and antioxidant enzymes, representing the body’s ability to fight oxidation (12, 34). The MDA is one of the lipid peroxidation products, and changes in MDA reflect the rate and intensity of lipid peroxidation in the body, and also indirectly reflect the degree of peroxidative damage in tissues (36). Wang et al. showed that GABA supplementation enhanced the antioxidant status of cows by reducing MDA accumulation in serum and increasing GSH-PX and SOD concentrations (12, 34). This is consistent with the study by Cheng et al. where cows fed RP-GABA had an enhanced antioxidant status and reduced oxidative stress under heat stress, as evidenced by a reduction in serum MDA and an increase in SOD, GSH-PX and T-AOC (17). However, in our study, GABA supplementation did not affect the serum concentrations of SOD, GSH-PX and T-AOC in cows and RP-GABA supplementation at 7.5 g/d promoted the accumulation of MDA. In contrast, on the time factor, as the experiment and lactation progressed, MDA accumulated in cow serum and SOD, GSH-PX and T-AOC levels decreased. Considering that the experimental herd maintained high lactation during the hot summer months, we suggest that the accumulation of MDA and the reduced levels of SOD, GSH-PX and T-AOC may be related to the lactation stress endured by the cows, but more data are needed to prove this.

Serum antioxidant levels in dairy cows can influence the level of inflammation in the organism, which is also related to the cow’s immunity. In previous studies, heat stress has led to a down-regulation of serum IgG and thus humoral immunity (37, 38). In addition, heat stress may also lead to inflammation, as evidenced by increased serum levels of IL-6 and TNF-α in cows (39). It is known that increased temperature leads to increased synthesis of HSP, and in particular HSP-70 expression is increased in heat-stressed cows, which has a protective effect against cellular stress (40–42). In our study, the addition of RP-GABA had no effect on IgG, IL-6, TNF-α and HSP-70 in cow serum, which is inconsistent with our hypothesis. Interestingly, on the time scale, levels of the inflammatory factors IL-6 and TNF, as well as HSP-70, which has an anti-stress effect, were upregulated in cow serum as the experiment progressed. Taking into account the changes in body antioxidant levels, we speculate that from 70 days to 120 days of lactation, cows with high yields also experience significant oxidative stress, which leads to the accumulation of inflammatory factors in their serum. Therefore, although cows maintain high-yielding performance despite heat stress, it is not a healthy state and we need to find new ways to promote cow health.

In summary, supplementing high-yielding cows with 10 g/d of RP-GABA during the hot summer months improved milk protein production but had no effect on other performance improvements, heat stress relief, immune function or antioxidant capacity. Although the results of this trial did not prove the initial hypothesis, given the excellent high yielding performance of the trial cows and the significant challenge of improving performance under heat stress, we believe that supplementation of 10 g/d RP-GABA to heat-stressed high yielding cows could provide some boost to performance. In conclusion, our study provides some reference for nutritional improvement measures for summer heat stress in dairy cows, especially high yielding cows.

## 5. Conclusion

In conclusion, this study shows that supplementing 10 g/d RP-GABA to early lactation cows in heat stress during the hot summer months can improve milk protein production, but has no effect on reducing heat stress, improving immune function or antioxidant capacity, which provides some nutritional options for the management of high yielding cows on summer farms.

## Data availability statement

The original contributions presented in the study are included in the article/supplementary material, further inquiries can be directed to the corresponding author.

## Ethics statement

The animal studies were approved by Yangzhou University, the Institutional Animal Care and Use Committee (SYXK (Su) IACUC 2016-0019). The studies were conducted in accordance with the local legislation and institutional requirements. Written informed consent was obtained from the owners for the participation of their animals in this study.

## Author contributions

YS, ZC, and ML conceptualized of research. ZC, WL, TW, and WW curated experimental data. WL and TW performed investigation for research. ZC, YS, TW, WL, and ML drafted writing-original manuscript. ZC and WW analyzed formal analysis. YS and ML presented methodology of research and offered resources. ZC and ML accomplished supervision for research. YS offered funding acquisition. All authors contributed to the article and approved the submitted version.

## Funding

This study was supported by the Sponsored by Shanghai Risingstar Program (NO.21QB1400300) and China Agriculture Research System (CARS-36).

## Conflict of interest

YS, TW, and WW are employed by Bright Farming Co. LTD.

The remaining authors declare that the research was conducted in the absence of any commercial or financial relationships that could be construed as a potential conflict of interest.

## Publisher’s note

All claims expressed in this article are solely those of the authors and do not necessarily represent those of their affiliated organizations, or those of the publisher, the editors and the reviewers. Any product that may be evaluated in this article, or claim that may be made by its manufacturer, is not guaranteed or endorsed by the publisher.
